# Performance of Xpert MTB/RIF and sputum microscopy compared to sputum culture for diagnosis of tuberculosis in seven hospitals in Indonesia

**DOI:** 10.3389/fmed.2022.909198

**Published:** 2023-01-20

**Authors:** Anis Karuniawati, Erlina Burhan, Eko Budi Koendhori, Desvita Sari, Budi Haryanto, Titik Nuryastuti, A. A. A. Yuli Gayatri, Uleng Bahrun, R. Lia Kusumawati, Retna Indah Sugiyono, Nugroho Harry Susanto, Aly Diana, Herman Kosasih, Adhella Menur Naysilla, Dewi Lokida, Aaron Neal, Sophia Siddiqui, Chuen-Yen Lau, Muhammad Karyana

**Affiliations:** ^1^Department of Microbiology, Faculty of Medicine, Universitas Indonesia, Dr. Cipto Mangunkusumo Hospital, Jakarta, Indonesia; ^2^Department of Pulmonary and Respiratory Medicine, Faculty of Medicine, Universitas Indonesia, Persahabatan Hospital, Jakarta, Indonesia; ^3^Department of Medical Microbiology, Faculty of Medicine, Universitas Airlangga, Dr. Soetomo Hospital, Surabaya, Indonesia; ^4^Department of Microbiology, Faculty of Medicine, Universitas Diponegoro, Dr. Kariadi Hospital, Semarang, Indonesia; ^5^Microbiology Unit, Persahabatan Hospital, Jakarta, Indonesia; ^6^Department of Microbiology, Faculty of Medicine, Public Health and Nursing, Universitas Gadjah Mada, Yogyakarta, Indonesia; ^7^Department of Internal Medicine, Faculty of Medicine, Universitas Udayana, Prof. IGNG. Ngoerah General Hospital, Bali, Indonesia; ^8^Department of Clinical Pathology, Faculty of Medicine, University of Hasanuddin, Dr. Wahidin Sudirohusodo Hospital, Makassar, Indonesia; ^9^Department of Microbiology, Faculty of Medicine, Universitas Sumatera Utara, H. Adam Malik General Hospital, Medan, Indonesia; ^10^Indonesia Research Partnership on Infectious Disease (INA-RESPOND), Jakarta, Indonesia; ^11^Department of Public Health, Faculty of Medicine, Universitas Padjadjaran, Sumedang, Indonesia; ^12^Department of Clinical Pathology, Tangerang District Hospital, Tangerang, Indonesia; ^13^National Institute of Allergy and Infectious Diseases, National Institutes of Health, Bethesda, MD, United States; ^14^HIV Dynamics and Replication Program, Center for Cancer Research, National Cancer Institute, National Institutes of Health, Bethesda, MD, United States; ^15^National Institute of Health Research and Development, Ministry of Health, Republic of Indonesia, Jakarta, Indonesia

**Keywords:** tuberculosis, diagnosis, sputum culture, sputum microscopy, Xpert MTB/RIF, DST, rifampicin, mycobacteria

## Abstract

**Introduction:**

Tuberculosis (TB) is a major public health concern in Indonesia, where the incidence was 301 cases per 100,000 inhabitants in 2020 and the prevalence of multi-drug resistant (MDR) TB is increasing. Diagnostic testing approaches vary across Indonesia due to resource limitations. Acid-fast bacilli (AFB) smear is widely used, though Xpert MTB/RIF has been the preferred assay for detecting TB and rifampicin resistance since 2012 due to higher sensitivity and ability to rapidly identify rifampicin resistance. However, <1,000 Xpert instruments were available in Indonesia as of 2020 and the Xpert supply chain has suffered interruptions.

**Methods:**

We compared the performance of Xpert MTB/RIF and AFB smear to facilitate optimization of TB case identification. We analyzed baseline data from a cohort study of adults with pulmonary TB conducted at seven hospitals across Indonesia. We evaluated sensitivity and specificity of AFB smear and Xpert MTB/RIF using *Mycobacterium tuberculosis* (Mtb) culture as the gold standard, factors associated with assay results, and consistency of Xpert MTB/RIF with drug susceptibility test (DST) in detecting rifampicin resistance.

**Results:**

Sensitivity of AFB smear was significantly lower than Xpert MTB/RIF (86.2 vs. 97.4%, *p*-value <0.001), but specificity was significantly better (86.7 vs. 73.3%, *p*-value <0.001). Performance varied by hospital. Positivity rate for AFB smear and Mtb culture was higher in subjects with pulmonary cavities and in morning sputum samples. Consistency of Xpert MTB/RIF with DST was lower in those with rifampicin- sensitive TB by DST.

**Discussion:**

Additional evaluation using sputa from primary and secondary Indonesian health centers will increase the generalizability of the assessment of AFB smear and Xpert MTB/RIF performance, and better inform health policy.

**Clinical trial registration:**

[https://clinicaltrials.gov/], identifier [NCT027 58236].

## Introduction

Tuberculosis (TB) is the most important public health problem in Indonesia. The TB incidence was 301 cases per 100,000 inhabitants in 2020 (1), second only to India globally (2). Indonesia is also amongst 30 nations designated as high multi-drug resistant (MDR)-TB burden countries (2, 3). Cases spread through 514 districts in 34 provinces across the 17,000 island archipelago (3). Geographic, economic and social diversity present challenges to standardization of healthcare practices, including TB diagnosis. Following the WHO recommendation, Xpert MTB/RIF became the front-line test for detection of TB and rifampicin resistance in 2012. However, <1,000 Xpert MTB/RIF instruments were available across 478 districts in 2020 (3). Thus, acid-fast bacilli (AFB) smear remains the standard at more than 12,000 primary and secondary healthcare facilities.

Though AFB smear sensitivity is relatively low (58–68% compared to sputum culture) (4), it is cost-effective, easy to perform, and offers rapid results (<1 h vs. 6–8 weeks for sputum culture) (5). Therefore, it is still widely used for diagnostic purposes and for monitoring treatment response. To overcome the limitations of AFB smear and sputum culture, Xpert MTB/RIF was endorsed by the WHO in 2010 (6). Xpert MTB/RIF is a rapid molecular-based assay which uses a single cartridge to simultaneously identify *Mycobacterium tuberculosis* (Mtb) and rifampicin resistance in 2 h. Performance has been evaluated in many studies that used varying designs and included different populations. Sensitivity ranges from 83 to 92% in Mtb culture-positive subjects and is estimated to be 67% in those who are AFB smear-negative; specificity ranges from 97 to 99% (7). However, the Xpert MTB/RIF instrument and cartridges are considered too expensive for broad implementation in lower and middle income countries and the supply chain has suffered interruptions in the last 2 years due to the switch of priority to COVID-19, disrupting TB case detection, prevention, and treatment (8).

We analyzed baseline data from a prospective cohort study of adults with pulmonary TB (TRIPOD) conducted at seven hospitals across Indonesia to assess the performance of AFB smear and Xpert MTB/RIF compared to sputum culture. Performance of Xpert MTB/RIF in AFB smear-negative but culture-positive pulmonary TB and factors associated with the positivity of AFB smear, Xpert MTB/RIF, and Mtb culture were also assessed.

We additionally evaluated the consistency of rifampicin resistance results from Xpert MTB/RIF and culture-based drug susceptibility test (DST) conducted at the five TB reference laboratories in Indonesia. These data are important for informing National TB Program (NTP) policy, diagnostic algorithms, prevention strategies, and management recommendations.

## Materials and methods

### Study design and population

This is a cross-sectional analysis of baseline data from a prospective cohort study that enrolled patients from February 2017 to November 2018 at seven DR-TB referral hospitals in seven large cities in Indonesia (Dr. Soetomo Hospital, Surabaya; Sanglah Hospital, Denpasar; Dr. Sardjito Hospital, Yogyakarta; Dr. Kariadi Hospital, Semarang; Persahabatan Hospital, Jakarta; H. Adam Malik Hospital, Medan; and Dr. Wahidin Sudirohusodo, Makassar). Enrollment criteria included age >18 years, presumptive pulmonary TB with cough for at least 2 weeks, at least one other TB symptom (fever, unexplained weight loss, loss of appetite, hemoptysis, shortness of breath, chest pain, night sweats, or fatigue) and consistent chest x-ray. Subjects were excluded if they had received TB treatment for more than 7 days in the last month, had any serious comorbidities (liver disease, chronic kidney disease, or psychiatric illness), or were pregnant at enrollment. Demographic, clinical, laboratory, and radiologic data were collected. Based on microbiological examinations (AFB smear, Xpert MTB/RIF, and culture), subjects were classified as (1) clinically diagnosed TB when none of the microbiological results were positive and (2) bacteriologically confirmed TB when at least one of the three examinations was positive. Clinicians were aware of all examination results and decided on the TB treatment after reviewing them.

### Specimen collection and testing

Sputum was obtained for AFB smear, Xpert MTB/RIF, and Mtb culture examination. Sputum was classified as a morning specimen if it was the first sputum produced for that date (overnight accumulation of secretions); sputum was otherwise classified as a spot specimen. Subjects were instructed to collect a minimum of 2 mL morning or spot sputum, which is sufficient for AFB smear, Xpert MTB/RIF, and culture. If the sputum was insufficient, separate specimens collected within 24 h were pooled together. While waiting for additional sputum, the earliest sputum was stored in the refrigerator at 2–8°C. Commonly, 2–3 sputa were pooled together. The same sputum was used for all examinations. Sputum was induced if necessary.

### Microbiological work-up

Acid-fast bacilli smear and Xpert MTB/RIF were performed at the study hospital microbiology laboratory. Mtb culture and DST were conducted at certified National TB Program Reference Laboratories and followed the hospital region’s standard of care. Adam Malik Hospital, Medan; Center for Health Laboratory, Surabaya; Center for Health Laboratory, Semarang; Clinical Microbiology Laboratory, Faculty of Medicine, University of Indonesia, Jakarta; Persahabatan Hospital, Jakarta; Sanglah Hospital, Denpasar; Microbiology Laboratory, UGM, Yogyakarta; Wahidin Sudirohusodo Laboratory, Makassar, and Hasanuddin University Medical Research Center, Makassar were the laboratories involved in this study.

### Acid-fast bacilli (AFB) smear microscopy test

Dedicated trained laboratory technicians conducted direct smear microscopy without sputum decontamination under supervision of a microbiologist. For direct smear microscopy, the laboratory technician used a loop to pick up a small amount (∼100 μL) of purulent sputum and transfer it to the slide. The smear was heat-fixed after being air-dried. The fixed smear was then flooded with carbol-fuchsin and gently heated to steaming for 5 min. The stain was then washed off with distilled water and decolorized with 3% acid-alcohol for 2–3 min. The acid-alcohol was washed off with distilled water, and the slide was tilted to drain. The slide was then flooded with methylene blue as a counterstain for 1–2 min before being washed with distilled water. After that, the slide was drained and air-dried. AFB remained red, whereas other organisms and cells took up the counterstain and turned blue. The laboratory technician examined the smear microscopically using the 100× oil immersion objective. Smear-positive specimens were reported semi-quantitatively (scanty, 1+, 2+, 3+, and 4+) using the standard scale from International Union Against TB and Lung Disease (IUATLD) as recommended by the United States–Centers for Disease Control and Prevention (US-CDC) (9).

### Xpert MTB/RIF

Xpert MTB/RIF was performed according to the manufacturer’s recommendations (Cepheid, Sunnyvale, CA, USA). Xpert MTB/RIF software was used to generate results.

### *Mycobacterium tuberculosis* culture and drug susceptibility test

Culture and DST methods varied amongst hospitals according to each national TB referral laboratories’ standards. Sputum was inoculated into solid media [Lowenstein Jensen (LJ) or Ogawa] prepared according to manufacturer’s instructions (BBL, BD, MD, USA) (10) or a liquid medium, Mycobacteria Growth Indicator Tube (BBL MGIT), which contains PANTA supplemented modified Middlebrook 7H9. Laboratories that used the LJ medium were Center for Health Laboratory, Surabaya; Center for Health Laboratory, Semarang; Sanglah Hospital, Denpasar; Microbiology Laboratory, UGM, Yogyakarta; and Wahidin Sudirohusodo Laboratory, Makassar. Persahabatan Hospital, Jakarta, used the Ogawa medium. Adam Malik Hospital, Medan; Clinical Microbiology Laboratory, Faculty of Medicine, University of Indonesia, Jakarta; and Hasanuddin University Medical Research Center, Makassar used the MGIT medium.

Before inoculation, sputum was decontaminated using a mixture of NALC (0.5%)–NaOH (2%) solution in equal volume. The mixing process was less than 30 s, and the mixture was then kept at room temperature for 15 min to decontaminate. The mixture was concentrated by centrifugation (3000 RPM for 15 min), then the supernatant was removed, and the sediment was resuspended in sterile phosphate buffer to a final volume of 2 ml. This suspension was then used for inoculation into LJ slant (2–3 drops) or BACTEC MGIT 960 culture tube (0.5 ml). Mtb was identified using TB Antigen MPT64 and para nitro-benzoic acid. The cultures were incubated at 35–37°C until growth was observed or discarded as negative after 8–10 weeks to anticipate a very slow growing Mtb.

Drug susceptibility test was performed with the MGIT system (Becton Dickinson, USA). We followed the WHO and Indonesia Ministry of Health TB control regulations available in 2016. These specified the concentration of drug in DST for first-line anti-TB drugs was to be 1.0 μg/ml Rifampicin, 0.1 μg ml Isoniazid, 5.0 μg/ml Ethambutol, and 1.0 μg/ml Streptomycin. The concentration of the drug in DST for second-line anti-TB drugs was to be 2 μg/ml Ofloxacin, 1 μg/ml Amikacin, and 1 μg/ml Kanamycin. According to this regulation, Pyrazinamide and other anti-TB drugs were not tested (11, 12).

### Statistical analysis

Descriptive data are presented as means (SD) and frequencies (percentages). Cases are categorized into new and previously treated TB cases based on subject report. Sensitivity and specificity for identification of Mtb were calculated for AFB smear and Xpert MTB/RIF using sputum culture as the gold standard. McNemar’s test was used to assess whether the sensitivity and specificity differ between AFB smear and Xpert MTB/RIF. Positivity rates of AFB smear, Xpert MTB/RIF, and sputum culture were analyzed based on gender, treatment history, the presence of cavities, and types of sputa using composite results of the three methods as the denominators. Sensitivity and specificity for detection of rifampicin susceptibility by Xpert MTB/RIF was calculated using DST as the gold standard.

### Study approvals

The Indonesia National Institute of Health and Research Development Health Research Ethics Committee provided ethical clearance for this study. The study has been registered at ClinicalTrials.gov with registration number: NCT02758236.

## Results

### Demography and case distribution by diagnostic method

**FIGURE 1:**
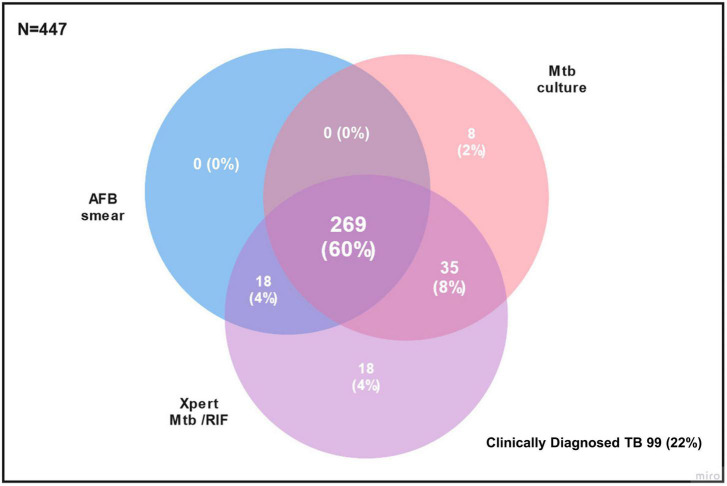
Case distribution by diagnostic methods. Number of participants are shown as n (%).

The TRIPOD study enrolled 490 subjects from 2017 to 2018. A total of 43 subjects were excluded for insufficient sputum (27 subjects), error or indeterminate Xpert MTB/RIF results (4 subjects), non-tuberculous mycobacteria or contaminated culture results (9 subjects), and no DST results (3 subjects). Of the 447 subjects, 312 subjects had positive sputum culture and were used for evaluation of sensitivity, the remaining 99 subjects that had no bacterial evidence of Mtb infection (AFB smear, Mtb culture, and Xpert MTB/RIF negative) and 36 subjects that were only AFB smear and/or Xpert MTB/RIF positive were used for evaluation of specificity. Of note, 33.1% (53/160) of negative AFB smear specimens were positive by Xpert MTB/RIF. Use of Xpert MTB/RIF added 53 cases (15.6%) of bacteriologically confirmed TB by AFB smear or Xpert MTB/RIF (340 subjects). Details are shown in Figure 1.

Mean (SD) subject age was 41.3 (14.1) years old, 274 (61.3%) subjects were male, and 260 (58.2%) cases were newly diagnosed TB. Kariadi and Adam Malik Hospitals contributed the most previously treated cases (83/187; 44.4%) (Table 1). The majority of subjects were from Soetomo, Surabaya (123), followed by Kariadi, Semarang (103), Persahabatan, Jakarta (80), and Sardjito, Yogyakarta (75).

**TABLE 1:** The distribution of sex, age, and treatment history in each study site.

	Sanglah	Wahidin	Kariadi	Soetomo	Sardjito	Persahabatan	Adam Malik	All
Enrolled subjects	32	25	108	128	83	89	25	490
Analyzed subjects	25	20	103	123	75	80	21	447
**Sex**								
Male	17 (68%)	13 (65%)	52 (51%)	71 (58%)	44 (59%)	63 (80%)	13 (62%)	274 (61%)
Female	8 (32%)	7 (35%)	51 (50%)	52 (42%)	31 (41%)	16 (20%)	8 (38%)	173 (39%)
Age (mean, SD year)	36.8 (12.2)	39.8 (17)	41.6 (12.2)	40.6 (12.8)	43.7 (17)	40.3 (14.7)	46.4 (15.1)	41.3 (14.1)
**Treatment history**								
Newly diagnosed	22 (88%)	19 (95%)	36 (35%)	70 (57%)	59 (79%)	49 (61%)	5 (24%)	260 (58%)
Previously treated	3 (12%)	1 (5%)	67 (65%)	53 (43%)	16 (21%)	31 (39%)	16 (76%)	187 (42%)

### Sensitivity and specificity of AFB smear and Xpert MTB/RIF compared to Mtb culture

The sensitivity of Xpert MTB/RIF was significantly higher than AFB smear (97.4 vs. 86.2%, *p* ≤ 0.001), while specificity was significantly lower (73.3 vs. 86.7%, *p* < 0.001) (Table 2). The sensitivities of AFB smear and Xpert MTB/RIF in newly diagnosed subjects were comparable to previously treated subjects (83.8 vs. 89.2%, *p* = 0.17, and 97.1 vs. 97.8%, *p* = 0.73, respectively). However, AFB smear specificity was significantly higher in newly diagnosed subjects, while that for Xpert MTB/RIF trended toward being higher though this was not statistically significant (92 vs. 77.1%, *p* = 0.01, and 77 vs. 66.7%, *p* = 0.19, respectively). Details of the AFB smear and Xpert MTB/RIF sensitivity and specificity analysis, including performance by site, are shown in Table 2.

**TABLE 2:** Diagnosis of tuberculosis by AFB smear and Xpert MTB/RIF compared to sputum culture as the gold standard.

	AFB smear		Xpert MTB/RIF	
	**Sensitivity**	**Specificity**	**Sensitivity**	**Specificity**
All subjects	86.2% (269/312)	86.7% (117/135)	97.4% (304/312)	73.3% (99/135)
**History of treatment**				
New TB patients	83.8% (145/173)	92% (80/87)	97.1% (168/173)	77% (67/87)
Previously treated	89.2% (124/139)	77.1% (37/48)	97.8% (136/139)	66.7% (32/48)
**Sites/hospitals**				
Sanglah	68.8% (11/16)	100% (9/9)	87.5% (14/16)	88.9% (8/9)
Wahidin Sudirohusodo	83.3% (10/12)	100% (8/8)	91.7% (11/12)	62.5% (5/8)
Kariadi	90% (81/90)	76.9% (10/13)	100% (90/90)	61.5% (8/13)
Soetomo	94.3% (100/106)	64.7% (11/17)	96.2% (102/106)	52.9% (9/17)
Sardjito	50% (9/18)	93.0% (53/57)	100% (18/18)	84.2% (48/57)
Persahabatan	87.5% (49/56)	79.2% (19/24)	98.2% (55/56)	58.3% (14/24)
Adam Malik	64.3% (9/14)	100% (7/7)	100% (14/14)	100% (7/7)

Sensitivity of AFB smear was comparable amongst 5 sites (83.3–94.3%), but lower in Sardjito, Yogyakarta (50%) and Adam Malik, Medan (64.3%). Specificity ranged from 93 to 100% in four sites, and was lower at Soetomo, Surabaya (64.7%), Kariadi, Semarang (76.9%), and Persahabatan, Jakarta (79.2%). The sensitivity of Xpert MTB/RIF among sites was more consistent, with lowest sensitivity at Sanglah, Bali (87.5%) and highest at Kariadi, Semarang; Sardjito, Yogyakarta; and Adam Malik, Medan (100%). In contrast, specificity of Xpert MTB/RIF was only above 80% at two sites, Sanglah, Bali (88.9%) and Adam Malik, Medan (100%).

### Factors associated with the positivity of AFB smear, Xpert MTB/RIF, and Mtb culture

Table 3 compares the positivity of AFB smear, Xpert MTB/RIF, and Mtb culture by gender, treatment history, and presence of cavities in 348 subjects with bacteriologically confirmed TB. The positivity rate of AFB smear and Mtb culture was higher in subjects with cavities compared to those without cavities (89 vs. 76.1%, *p* = 0.002; 94.8 vs. 84.7%, *p* = 0.002). AFB smear positivity rate was also significantly higher in previously treated TB patients compared to newly diagnosed (87.1 vs. 78.8%, *p* = 0.04).

**TABLE 3:** The positivity of AFB smear, Xpert, and Mtb culture by presence of cavity, new and previous treatment, gender, and sputum type, compared to composite results of the three methods.

	AFB smear		Xpert MTB/RIF		Mtb culture	
Cavity	89 (153/172)	*p* = 0.002	99.4 (171/172)	*p* = 0.06	94.8 (163/172)	*p* = 0.002
No cavity	76.1 (134/176)		96 (169/176)		84.7 (149/176)	
Newly diagnosed TB	78.8 (152/193)	*p* = 0.04	97.4 (188/193)	0.73	89.6 (173/193)	*p* = 0.99
Previously treated TB	87.1 (135/155)		98.1 (152/155)		89.7 (139/155)	
Male	81.2 (177/218)	*p* = 0.41	98.6 (215/218)	*p* = 0.13	87.2 (190/218)	*p* = 0.04
Female	84.6 (110/130)		96.2 (125/130)		93.9 (122/130)	
Morning sputum	85.4 (204/239)	*p* = 0.04	98.7 (236/239)	*p* = 0.33	90.8 (217/239)	*p* = 0.07
Mixed sputum	89.8 (44/49)		98 (48/49)		81.6 (40/49)	
Spot sputum	74.2 (49/66)		98.5 (65/66)		83.3 (55/66)	

*Mycobacterium tuberculosis* culture using morning sputum vs. mixed or spot trended toward being more positive, though differences did not achieve significance. For AFB smear, the positivity rate was significantly different with highest positivity rate in mixed sputum and lowest rate in spot sputum (*p* = 0.04). Type of sputum did not affect the positivity of Xpert MTB/RIF.

### Rifampicin resistance

Table 4 shows that the consistency of Xpert MTB/RIF with DST was better in the rifampicin-resistant group than in the rifampicin-sensitive group (96 vs. 84%). Six rifampicin-sensitive and two rifampicin-resistant sputa were negative by Xpert MTB/RIF. Consistency of Xpert MTB/RIF in rifampicin-resistant cases across sites was excellent. In the rifampicin sensitive group, Xpert MTB/RIF classified 50, 15, and 12.5% as resistant in Kariadi, Semarang, Soetomo, Surabaya, and Sanglah, Bali, respectively.

**TABLE 4:** Performance of Xpert MTB/RIF vs. DST for rifampicin susceptibility testing by sites/hospitals.

	Positive Mtb culture (*n* = 312)					
**DST**	**Rifampicin sensitive (*n* = 196)**			**Rifampicin resistant (*n* = 116)**		
**Xpert MTB/RIF**	**Negative *n* (%)**	**Sensitive *n* (%)**	**Resistant *n* (%)**	**Negative *n* (%)**	**Sensitive *n* (%)**	**Resistant *n* (%)**
**sites/hospitals**						
Sanglah	2 (12.5)	12 (75)	2 (12.5)	0 (0)	0 (0)	0 (0)
Wahidin Sudirohusodo	1 (9)	10 (91)	0 (0)	0 (0)	0 (0)	1 (100)
Kariadi	0 (0)	10 (50)	10 (50)	0 (0)	1 (1)	69 (99)
Soetomo	2 (2)	67 (83)	12 (15)	2 (8)	0 (0)	23 (92)
Sardjito	0 (0)	14 (100)	0 (0)	0 (0)	0 (0)	4 (100)
Persahabatan	1 (2)	48 (96)	1 (2)	0 (0)	1 (17)	5 (83)
Adam Malik	0 (0)	4 (100)	0 (0)	0 (0)	0 (0)	10 (100)
Total	6 (3)	165 (84)	25 (13)	2 (2)	2 (2)	112 (96)

## Discussion

In this prospective cohort of adults with TB conducted at seven major Indonesian hospitals, we enrolled newly diagnosed and previously treated TB cases and assessed drug-susceptibility profiles. The sensitivity of Xpert MTB/RIF was significantly higher than that of AFB smear while specificity was significantly lower when Mtb culture was considered the gold standard. Xpert MTB/RIF demonstrated good sensitivity at all sites, while other performance characteristics for Xpert MTB/RIF and AFB smear showed high variation. Furthermore, higher positivity rates of AFB smear and Mtb culture were found in subjects with cavities and in morning sputum samples. Lastly, discordance between Xpert MTB/RIF and Mtb culture DST results mainly occurred in rifampicin sensitive sputum by DST.

Our findings confirm previous reports that Xpert MTB/RIF is better than AFB smear for detection of TB (13, 14). A review of 48 high quality studies showed that Xpert MTB/RIF has a sensitivity of 96% (95% CI: 94–97%) (15). Xpert MTB/RIF detects the genome of Mtb rather than the whole bacilli detected by AFB smear, so would be expected to be more sensitive (6). Furthermore, interpretation of AFB smear requires training and is more subjective, particularly in sputa with lower bacilli load (16). In our study, overall sensitivity of AFB smear, though lower than Xpert MTB/RIF, was still higher than in other reports. Our subjects were more likely to be AFB smear positive because 45% of them were previously treated, and the new TB cases were likely more complicated as they were already being seen at our TB referral study sites (more problematic than subjects at the primary health centers). The low sensitivity of AFB smear at two study sites (Sardjito and Adam Malik hospitals) may be due to a high proportion of newly diagnosed subjects with no pulmonary cavities, suggesting low bacillary load that resulted in negative AFB smear.

The low specificity of Xpert MTB/RIF compared to Mtb culture has been reported previously (17, 18). Similar to our study, the low specificity in these two studies was due to the inclusion of re-treated TB patients. Therefore, our results are representative of centers with similar populations. Performance may be different in centers where newly diagnosed TB patients predominate. One plausible explanation is that Xpert MTB/RIF detects residual genomes in previously treated subjects. Persistent detection 5 years after pulmonary TB treatment has been reported (19, 20) with up to 27% of patients remaining positive 6 months after successful treatment (21, 22). Theron et al. reported that patients false-positive by Xpert MTB/RIF were more likely to have recent previous TB with low Mtb DNA load (high Ct value), and chest X-ray non-suggestive of active-TB (23). Accordingly, our study showed the specificity of Xpert MTB/RIF trended toward being higher in newly diagnosed subjects, though it was not statistically significant. Thus, a thorough exploration of clinical, radiologic and TB treatment history, in conjunction with detailed Xpert MTB/RIF results should be undertaken for patients who are Xpert MTB/RIF positive and culture negative (18). Additionally, the low specificity of Xpert MTB/RIF, suggests that AFB smear still has a role, particularly during monitoring of treatment response (22). However, positive Xpert MTB/RIF but negative culture can be seen in newly diagnosed patients with low cavitation frequency and low bacillary load (24).

The high sensitivity of Xpert MTB/RIF (98.4%) compared to AFB smear in our study highlights the potential benefit of broadly deploying Xpert MTB/RIF. For case identification in high prevalence settings, the more sensitive assay should be used, even at the cost of specificity. Xpert MTB/RIF is available in only about 1,000 healthcare facilities in Indonesia. Capacity should be scaled up as a component of public health policy to combat TB. Expansion of Xpert MTB/RIF testing to district hospitals, primary healthcare facilities, and private providers must be accompanied by training and resources to support sustainability (25). Use of Xpert MTB/RIF can enhance bacteriologic confirmation of TB, improve detection of rifampicin resistance, shorten the time to diagnosis, reduce transmission, and enable early treatment (6). Despite the benefit of detecting rifampicin resistance in just a few hours by Xpert MTB/RIF, rifampicin resistance results should be interpreted with caution as the phenotype may still be sensitive.

Our finding that AFB smear and Mtb culture are more sensitive in subjects with cavities is consistent with a prior study comparing bacillary loads between TB patients with and without cavities. Mean log_10_ CFU/ml of sputum values for cavitary (*n* = 100) and non-cavitary (*n* = 144) patients, were 5.2 ± 1.4 (range, 1.1–7.3) and 4.0 ± 1.6 (range, 0.9–7.4), respectively (*P* ≤ 0.0001) (26). However, we found a subset of smear positive but culture negative samples. Technical issues such as improper sputum storage and transportation, inaccurate decontamination and centrifugation procedures, and temperature fluctuations in the incubator might have contributed to lack of culture growth and the requirement for repeat culture testing (27). A biological explanation for failure to detect growth of the Mtb culture is the presence of non-replicating bacteria, and mycobacterial resuscitation-promoting factor (RPF) treatment may activate the growth of dormant bacteria (28). Pang Y et al. reported 5.7% growth detection failure of BACTEC MGIT 960 culture reflected Mtb poor fitness *in vitro*, despite the positivity grade of AFB smear (29).

Our trend toward better sensitivity of AFB smear and Mtb culture with morning sputum aligns with a systematic review and meta-analysis that showed early morning sputum was superior to other sputum specimens, though the difference was not significant (OR 1⋅5, 95% CI 0⋅9–2⋅6, *p* = 0⋅2, for AFB smear; and 1⋅4, 0⋅9–2⋅4, *p* = 0⋅2, for Mtb culture) (30). Concentration of bacilli in early morning samples may be due to accumulation of sputum in the lungs overnight (31). As for Xpert MTB/RIF, the very sensitive nature of this assay suggests no specific sputum is preferred (32).

We found that discordance between Xpert MTB/RIF and Mtb culture DST mostly occurred in sputum rifampicin sensitive by DST, but resistant by Xpert MTB/RIF (25 of 27 discordant cases). It has been reported that DST in South Africa identifies less than 50% of rifampicin resistance compared to Xpert MTB/RIF (33). Similar results have also been reported in Iran and South Africa, regardless of HIV status (34–36). This discordance between Xpert MTB/RIF and DST is most likely due to the ability of Xpert MTB/RIF to detect silent mutations in which the genetic changes have no impact on protein structure (37). Also, Xpert MTB/RIF can detect borderline rifampicin resistance, which is associated with low levels of rifampicin resistance (38). However, very low bacillary loads may result in insufficient amplification of sequences present on specific Xpert MTB/RIF probes, resulting in attachment failure and false-positive rifampicin resistance results (39). Absent or delayed specific probes, such as B or E, are also associated with false-positive rifampicin resistance by Xpert MTB/RIF (40). Amongst our 25 discordant cases, 10 were newly diagnosed and 15 were previously treated. Following the national TB guideline, sputa from the 10 newly diagnosed subjects were re-tested for Xpert MTB/RIF; one of these subjects was rifampicin-sensitive, treated as DS-TB and cured. Seven newly diagnosed subjects who were treated as MDR-TB were also cured, whereas two others were lost to follow-up.

The two subjects with rifampicin sensitivity by Xpert MTB/RIF but phenotypic resistance by DST included one newly diagnosed and one previously treated subject. The newly diagnosed subject was treated as DS-TB even after the DST revealed resistance to rifampicin. However, the treatment was extended through 1 year. Treatment for the previously treated subject was switched from DS-TB to DR-TB once the DST results that showed rifampicin, INH, ethambutol, streptomycin, and ofloxacin resistance were available. Both subjects were cured. The discordance of Xpert MTB/RIF and DST in these two patients may have several explanations. Xpert MTB/RIF cannot detect rifampicin resistance unless 65–100% of the DNA population in the sample is mutant (41). Failure in detecting several mutations by Xpert MTB/RIF may occur due to binding competition if the mutation is located at the probe junctions (42). The rare (2–5%) rpoB mutations can also occur outside the rifampicin-resistant determining region (RRDR), which has been reported by two studies in Indonesia. In Makassar, South Sulawesi, 80% (40/50) of Mtb isolates had these mutations (Gln432Pro, Asp435Val, Ser441Leu, His445Asp, Ser450Leu, and Ile491Phe). In Jayapura, Papua, 89% (17/19) of Mtb isolates had these mutations (Leu430Pro, Gln432Lys, Asp435Tyr, His445Tyr, and Ser450Leu) (43, 44).

In addition, the high prevalence (1.9–28.8%) of hetero-resistant sputum, which contains both sensitive and resistant Mtb bacilli in regions with high TB endemicity, may result in rifampicin resistance by Xpert MTB/RIF but sensitive in sputum culture, or vice versa (45, 46). This may explain the high discordance of results from two TB referral hospitals (Kariadi and Soetomo) in Central and East Java provinces where TB cases were most prevalent in Indonesia (1). As these two hospitals enrolled many previously treated patients, a higher proportion of mixed-type bacilli and rare mutations would be expected (47). Almost all plausible explanations above are biological, therefore we cannot determine whether results from one of the assays was inaccurate. Clinicians should carefully assess results from both assays to avoid under- and over-diagnosis of rifampicin resistant TB cases. Regarding the rare mutations outside the RRDR that are missed by XPert MTB/RIF, improvement is needed. Finally, as the epidemiology of rpoB mutations in Indonesia is still limited, further study to evaluate the performance of the Xpert MTB/RIF for detecting rifampicin resistance (48) is required, including gene amplification and sequence analysis.

Our study had several limitations. First, it was conducted in the referral hospital setting in large cities, which have fewer supply chain and human resource limitations. Use of Xpert MTB/RIF may not be feasible in more remote settings. Furthermore, we found high performance variation across sites. Both the high variation and specific population mean that our results cannot be generalized to other types of healthcare facilities. However, our results show that AFB smear performs reasonably well and still has a role in TB control efforts. Lastly, our population was skewed toward previously treated subjects and problematic new subjects, which may have affected performance measures.

Our findings support the need to expand and decentralize Xpert MTB/RIF for TB case detection, particularly in newly diagnosed TB, to improve TB control in Indonesia. Though Xpert MTB/RIF aids in detecting rifampicin resistance, clinicians should carefully evaluate results on a case-by-case basis and consider DST results. We recommend maintaining the capacity for AFB smear for monitoring treatment response as it demonstrated higher specificity than Xpert MTB/RIF, is relatively easy to perform, and is inexpensive. Further studies should assess strategies for scale-up of Xpert MTB/RIF, evaluate performance of TB diagnostics in remote areas, and explore strategies to improve consistency amongst Xpert MTB/RIF, AFB smear, and DST.

## Data availability statement

The raw data supporting the conclusions of this article will be made available by the authors, without undue reservation.

## Ethics statement

The studies involving human participants were reviewed and approved by the Indonesia National Institute of Health and Research Development Health Research Ethics Committee. The patients/participants provided their written informed consent to participate in this study.

## Author contributions

AK, EB, RS, NS, HK, and SS: study conception and design. EK, DS, BH, TN, AG, UB, and RK: data collection. AK, EB, RS, NS, HK, DL, C-YL, AN, SS, and MK: analysis and interpretation of results. AK, EB, RS, NS, AD, HK, AMN, C-YL, AN, and SS: draft manuscript preparation. All authors reviewed the results and approved the final version of manuscript.

## References

[1] Ministry of Health Republic Indonesia. *Indonesian Health Profile 2020.* Jakarta: Ministry of Health Republic Indonesia (2021).

[2] World Health Organization. *Global Tuberculosis Report 2020.* Geneva: World Health Organization (2020).

[3] Joint External Monitoring Mission. *The Republic of Indonesia - Joint External Monitoring Mission for Tuberculosis.* Jakarta: JEMM (2020).

[4] Davis J Cattamanchi A Cuevas L Hopewell P Steingart K. Diagnostic accuracy of same-day microscopy versus standard microscopy for pulmonary tuberculosis: a systematic review and meta-analysis. *Lancet Infect Dis.* (2013) 13:147–54. 10.1016/S1473-3099(12)70232-3 23099183 PMC3836432

[5] Bayot ML Mirza TM Sharma S. *Acid Fast Bacteria.* Treasure Island, FL: StatPearls Publishing (2021). 30725806

[6] World Health Organization. *Xpert MTB/RIF Implementation Manual – Technical and Operational ‘How-to’: Practical Considerations.* Geneva: WHO (2014). 25473699

[7] Steingart K Sohn H Schiller I Kloda L Boehme C Pai M Xpert^®^ MTB/RIF assay for pulmonary tuberculosis and rifampicin resistance in adults. *Cochrane Database Syst Rev.* (2013) 1:CD009593. 10.1002/14651858.CD009593.pub2 PMC4470352 23440842

[8] World Health Organization. *Global Tuberculosis Report 2021.* Geneva: World Health Organization (2021).

[9] Centers for Disease Control and Prevention. *Diagnosis of Tuberculosis Disease.* Atlanta, GA: CDC (0000).

[10] Becton, Dickinson and Company. *BBLTM Lowenstein-Jensen Medium BBLTM Lowenstein-Jensen Medium with 5% Sodium Chloride.* Sparks, MD: Becton, Dickinson and Company (2015).

[11] World Health Organization. *Definitions and Reporting Framework for Tuberculosis – 2013 Revision (Updated December 2014 and January 2020).* Geneva: World Health Organization (2013).

[12] Menteri Kesehatan Republik Indonesia. *Peraturan Menteri Kesehatan Republik Indonesia nomor 67 tahun 2016 tentang Penanggulangan Tuberkulosis.* (2016). Available online at: http://hukor.kemkes.go.id/uploads/produk_hukum/PMK_No._67_ttg_Penanggulangan_Tuberkolosis_.pdf (accessed November 9, 2022).

[13] Rice J Seifert M Moser K Rodwell T. Performance of the Xpert MTB/RIF assay for the diagnosis of pulmonary tuberculosis and rifampin resistance in a low-incidence, high-resource setting. *PLoS One.* (2017) 12:e0186139. 10.1371/journal.pone.0186139 29016684 PMC5633176

[14] Li S Lin L Zhang F Zhao C Meng H Wang H. A retrospective study on Xpert MTB/RIF for detection of tuberculosis in a teaching hospital in China. *BMC Infect Dis.* (2020) 20:362. 10.1186/s12879-020-05004-8 32448123 PMC7245878

[15] Horne D Kohli M Zifodya J Schiller I Dendukuri N Tollefson D Xpert MTB/RIF and Xpert MTB/RIF ultra for pulmonary tuberculosis and rifampicin resistance in adults. *Cochrane Database Syst Rev.* (2019) 6:CD009593. 10.1002/14651858.CD009593.pub4 31173647 PMC6555588

[16] Reji P Aga G Abebe G. The role of AFB microscopy training in improving the performance of laboratory professionals: analysis of pre and post training evaluation scores. *BMC Health Serv Res.* (2013) 13:392. 10.1186/1472-6963-13-392 24099153 PMC3851756

[17] Meawed T Shaker A. Assessment of diagnostic accuracy of Gene Xpert MTB/RIF in diagnosis of suspected retreatment pulmonary tuberculosis patients. *Egypt J Chest Dis Tuberc.* (2016) 65:637–41. 10.1016/j.ejcdt.2016.04.005

[18] Haraka F Schumacher S Ross A Mantsoki A Gagneux S Reither K Effect of history of tuberculosis on specificity of Xpert MTB/RIF. *Eur Respir J.* (2020) 56:2000343. 10.1183/13993003.00343-2020 32409573

[19] Boyles T Hughes J Cox V Burton R Meintjes G Mendelson M. False-positive Xpert^®^ MTB/RIF assays in previously treated patients: need for caution in interpreting results. *Int J Tuberc Lung Dis.* (2014) 18:876–8. 10.5588/ijtld.13.0853 24902569

[20] Metcalfe J Makumbirofa S Makamure B Mutetwa R Peñaloza R Sandy C Suboptimal specificity of Xpert MTB/RIF among treatment-experienced patients. *Eur Respir J.* (2015) 45:1504–6. 10.1183/09031936.00214114 25792637 PMC4948946

[21] Nicol M. Xpert MTB/RIF: monitoring response to tuberculosis treatment. *Lancet Respir Med.* (2013) 1:427–8. 10.1016/S2213-2600(13)70133-4 24429228

[22] Friedrich S Rachow A Saathoff E Singh K Mangu C Dawson R Assessment of the sensitivity and specificity of Xpert MTB/RIF assay as an early sputum biomarker of response to tuberculosis treatment. *Lancet Respir Med.* (2013) 1:462–70. 10.1016/S2213-2600(13)70119-X 24429244

[23] Theron G Venter R Calligaro G Smith L Limberis J Meldau R Xpert MTB/RIF results in patients with previous tuberculosis: can we distinguish true from false positive results? *Clin Infect Dis.* (2016) 62:995–1001. 10.1093/cid/civ1223 26908793 PMC4803105

[24] Nguyen M Jenny-Avital E Burger S Leibert E Achkar J. Clinical and radiographic manifestations of sputum culture-negative pulmonary tuberculosis. *PLoS One.* (2015) 10:e0140003. 10.1371/journal.pone.0140003 26448182 PMC4598139

[25] Albert H Nathavitharana R Isaacs C Pai M Denkinger C Boehme C. Development, roll-out and impact of Xpert MTB/RIF for tuberculosis: what lessons have we learnt and how can we do better? *Eur Respir J.* (2016) 48:516–25. 10.1183/13993003.00543-2016 27418550 PMC4967565

[26] Palaci M Dietze R Hadad D Ribeiro F Peres R Vinhas S Cavitary disease and quantitative sputum bacillary load in cases of pulmonary tuberculosis. *J Clin Microbiol.* (2007) 45:4064–6. 10.1128/JCM.01780-07 17928422 PMC2168542

[27] Mnyambwa N Ngadaya E Kimaro G Kim D Kazwala R Petrucka P Assessment of sputum smear-positive but culture-negative results among newly diagnosed pulmonary tuberculosis patients in Tanzania. *Int J Gen Med.* (2017) 10:199–205. 10.2147/IJGM.S137469 28744153 PMC5513826

[28] Dusthackeer A Balasubramanian M Shanmugam G Priya S Nirmal C Sam Ebenezer R Differential culturability of mycobacterium tuberculosis in culture-negative sputum of patients with pulmonary tuberculosis and in a simulated model of dormancy. *Front Microbiol.* (2019) 10:2381. 10.3389/fmicb.2019.02381 31749768 PMC6842972

[29] Pang Y Su B Zheng H Zhang Z Ma A Wang Y Factors associated with missed detection of mycobacterium tuberculosis by automated BACTEC MGIT 960 system. *Biomed Res Int.* (2016) 2016:1–4. 10.1155/2016/5972021 28078294 PMC5204086

[30] Datta S Shah L Gilman R Evans C. Comparison of sputum collection methods for tuberculosis diagnosis: a systematic review and pairwise and network meta-analysis. *Lancet Glob Health.* (2017) 5:e760–71. 10.1016/S2214-109X(17)30201-2 28625793 PMC5567202

[31] Ssengooba W Kateete D Wajja A Bugumirwa E Mboowa G Namaganda C An early morning sputum sample is necessary for the diagnosis of pulmonary tuberculosis, even with more sensitive techniques: a prospective cohort study among adolescent TB-suspects in Uganda. *Tuberc Res Treat.* (2012) 2012:1–6. 10.1155/2012/970203 23304491 PMC3529437

[32] Lee H Kee S Shin J Kwon Y Chun S Lee J Xpert MTB/RIF assay as a substitute for smear microscopy in an intermediate-burden setting. *Am J Respir Crit Care Med.* (2019) 199:784–94. 10.1164/rccm.201804-0654OC 30252496

[33] Kenaope L Ferreira H Seedat F Otwombe K Martinson N Variava E. Sputum culture and drug sensitivity testing outcome among X-pert Mycobacterium tuberculosis/rifampicin-positive, rifampicin-resistant sputum: a retrospective study — Not all rifampicin resistance is multi-drug resistant. *J Glob Antimicrob Resist.* (2020) 21:434–8. 10.1016/j.jgar.2019.11.008 31733411

[34] Nasiri M Zamani S Pormohammad A Feizabadi M Aslani H Amin M The reliability of rifampicin resistance as a proxy for multidrug-resistant tuberculosis: a systematic review of studies from Iran. *Eur J Clin Microbiol Infect Dis.* (2018) 37:9–14. 10.1007/s10096-017-3079-4 28823010

[35] Dlamini-Mvelase N Werner L Phili R Cele L Mlisana K. Effects of introducing Xpert MTB/RIF test on multi-drug resistant tuberculosis diagnosis in KwaZulu-Natal South Africa. *BMC Infect Dis.* (2014) 14:442. 10.1186/1471-2334-14-442 25129689 PMC4141089

[36] Osman M Simpson J Caldwell J Bosman M Nicol M. GeneXpert MTB/RIF version G4 for identification of rifampin-resistant tuberculosis in a programmatic setting. *J Clin Microbiol.* (2014) 52:635–7. 10.1128/JCM.02517-13 24478501 PMC3911341

[37] Ajbani K Kazi M Tornheim J Naik S Soman R Shetty A Pyrosequencing to resolve discrepant Xpert MTB/RIF and mycobacterial growth indicator tube 960. *Lung India.* (2018) 35:168–70. 10.4103/lungindia.lungindia_71_17 29487256 PMC5846270

[38] Xia H Song Y Zheng Y Wang S Zhao B He W Detection of Mycobacterium tuberculosis rifampicin resistance conferred by borderline rpoB mutations: Xpert MTB/RIF is superior to phenotypic drug susceptibility testing. *Infect Drug Resist.* (2022) 15:1345–52. 10.2147/IDR.S358301 35378895 PMC8976515

[39] Ngabonziza J Decroo T Migambi P Habimana Y Van Deun A Meehan C Prevalence and drivers of false-positive rifampicin-resistant Xpert MTB/RIF results: a prospective observational study in Rwanda. *Lancet Microbe.* (2020) 1:e74–83. 10.1016/S2666-5247(20)30007-0 35544156

[40] Berhanu R Schnippel K Kularatne R Firnhaber C Jacobson K Horsburgh C Discordant rifampicin susceptibility results are associated with Xpert ^®^ MTB/RIF probe B and probe binding delay. *Int J Tuberc Lung Dis.* (2019) 23:358–62. 10.5588/ijtld.16.0837 30940300 PMC6495054

[41] Blakemore R Story E Helb D Kop J Banada P Owens M Evaluation of the analytical performance of the Xpert MTB/RIF assay. *J Clin Microbiol.* (2010) 48:2495–501. 10.1128/JCM.00128-10 20504986 PMC2897495

[42] Singh U Pandey P Mehta G Bhatnagar A Mohan A Goyal V Genotypic, phenotypic and clinical validation of genexpert in extra-pulmonary and pulmonary tuberculosis in India. *PLoS One.* (2016) 11:e0149258. 10.1371/journal.pone.0149258 26894283 PMC4760939

[43] Umar F Husain D Hatta M Natzir R Sjahril R Dwiyanti R Molecular characterisation of mutations associated with resistance to first- and second-line drugs among Indonesian patients with tuberculosis. *J Taibah Univ Med Sci.* (2020) 15:54–8. 10.1016/j.jtumed.2019.12.003 32110183 PMC7033412

[44] Maladan Y Krismawati H Wahyuni T Tanjung R Awaludin K Audah K The whole-genome sequencing in predicting *Mycobacterium tuberculosis* drug susceptibility and resistance in Papua, Indonesia. *BMC Genomics.* (2021) 22:844. 10.1186/s12864-021-08139-3 34802420 PMC8607662

[45] Zetola N Shin S Tumedi K Moeti K Ncube R Nicol M Mixed *Mycobacterium tuberculosis* complex infections and false-negative results for Rifampin resistance by GeneXpert MTB/RIF are associated with poor clinical outcomes. *J Clin Microbiol.* (2014) 52:2422–9. 10.1128/JCM.02489-13 24789181 PMC4097703

[46] Rando-Segura A Aznar M Moreno M Espasa Soley M Sulleiro Igual E Bocanegra Garcia C Molecular characterization of rpoB gene mutations in isolates from tuberculosis patients in Cubal, Republic of Angola. *BMC Infect Dis.* (2021) 21:1056. 10.1186/s12879-021-06763-8 34641802 PMC8507306

[47] Fang R Li X Li J Wu J Shen X Gui X Mixed infections of *Mycobacterium tuberculosis* in tuberculosis patients in Shanghai, China. *Tuberculosis.* (2008) 88:469–73. 10.1016/j.tube.2008.02.002 18424179 PMC4391511

[48] Williamson D Basu I Bower J Freeman J Henderson G Roberts S. An evaluation of the Xpert MTB/RIF assay and detection of false-positive rifampicin resistance in Mycobacterium tuberculosis. *Diagn Microbiol Infect Dis.* (2012) 74:207–9. 10.1016/j.diagmicrobio.2012.06.013 22819240

